# Combined Endodontic and Surgical Management of Twin Rooted Maxillary Lateral Incisor with a Palatogingival Groove

**DOI:** 10.22037/iej.v13i3.21366

**Published:** 2018

**Authors:** Arshad Hasan, Javeria Ali Khan

**Affiliations:** a *Department of Operative Dentistry, Dow Dental College, Dow University of Health Sciences, Baba-e-Urdu Road Karachi, Pakistan *

**Keywords:** Aberrant Anatomy, Accessory Root, Maxillary Lateral Incisor, Retreatment, Two Roots

## Abstract

A case of unusual anatomy in a maxillary lateral incisor is presented. A 20-year old female presented with failing endodontics. Clinical examination and radiographs revealed poorly obturated maxillary left lateral incisor with an untreated patent second root and a palatogingival groove. A decision was made to treat the tooth non-surgically. After removing gutta-percha from main canal, the orifice of second root could not be located from inside the chamber. After determining the position of this root to be mesial and palatal to main canal, gingival tissue was removed from mesio-palatal side and access was extended to include the cingulum and orifice was located mesio-palatally. This canal was mechanically prepared and both canals were filled with calcium hydroxide. Three weeks later when symptoms subsided, the canals were obturated using the warm vertical technique. However, patient returned after a month complaining of pain and pus discharge. The tooth was surgically retreated; the enucleation was performed without root end resection. Patient returned after two years and reported complete healing but with mild discoloration of crown which was treated with walking bleach technique.

## Introduction

Knowledge of normal and aberrant root canal anatomy is a pre-requisite for successful outcome of endodontic treatment. Maxillary lateral incisor has a high incidence of pulpal anatomy variations most common of which are palatogingival groove (PGG) and dens invaginatus [1]. The presence of more than one canal has also been reported but two separate roots in this tooth with a concomitant palatogingival groove are rare and only occasionally reported previously. It has been suggested that presence of PGG may be an attempt by the Hertwig’s Epithelial Root Sheath (HERS) to form a separate root [2, 3]. The reported incidence of PGG in maxillary lateral incisor is 3%, while the incidence of two separate roots in maxillary lateral incisor is not known [4]. A literature search on PubMed revealed 29 case reports (33 cases) of two separate roots in this tooth, with each report suggesting different treatment options (Table 1) [3, 5-32]. The smaller, accessory root was either retained and root can be treated or extracted. The decision to retain or extract the root depends on many variables most important of which are patency of the canal, presence of palatogingival groove with concomitant periodontal defect and esthetics. Esthetics may be compromised if removal of this root results in loss of papilla due to loss of interproximal bone between central and lateral incisor [33]. On the other hand, removal may be necessary to allow for orthodontic tooth alignment [31]. This case report presents non-surgical and surgical re-treatment of bi-rooted maxillary lateral incisor, its clinical, radiographic features and its follow-up after 2 years. In the absence of three dimensional imaging, the guess work of canal location resulted in sacrificing of part of cingulum and overlying gingiva since the orifice was found outside the pulp chamber. This report also describes the 2-year follow-up of the case and internal bleaching that was performed to correct discoloration of tooth on follow-up.

## Case Report

**Figure 1:**
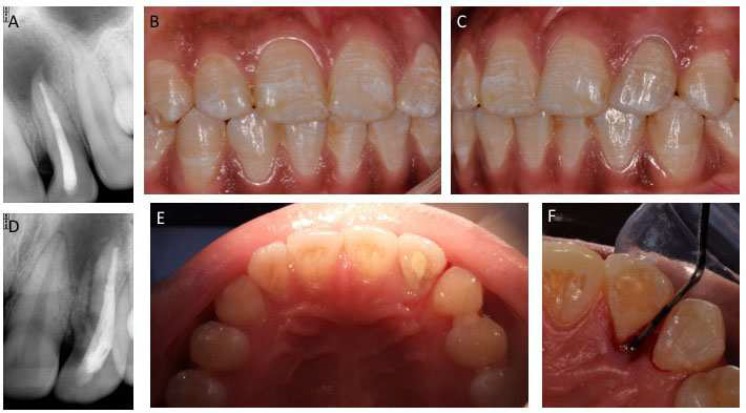
*A)* Pre-operative Radiograph with a periapical radiolucency and a hint of additional root; *B)* Frontal view of right maxillary incisor; *C)* Frontal view of left maxillary incisor; it was longer occlusogingivally and wider mesiodistally than its contralateral; *D)* Mesial shift view revealed the accessory root and associated radiolucency; *E)* Palatogingival groove is present below cingulum; *F)* A periodontontal defect of 5 mm

A 20 year-old female patient was referred to the Department of Operative Dentistry, Dow Dental College, complaining of pain in a previously treated left maxillary lateral incisor. Clinically there was pain on percussion without a discharging sinus and normal mobility. The clinical crown was wider than the contralateral with an unusual cervical depression mesially (Figure 1B and C). Periodontal examination (CP 12 periodontal probe, Hu Friedy) revealed a 5 mm isolated probing defect on the palatal aspect with an associated shallow palatogingival groove (Figure 1F). Cold test (Roeko Endo Frost, Coltene/Whaledent Pvt Ilt, Mumbai India) revealed that the lateral incisor was non vital, while the adjacent central incisor and canine were vital. Radiographically, there was poorly obturated canal associated with a periapical radiolucency (periapical index score 5) and an untreated accessory root with a patent canal (Figures 1A and D) [34]. A diagnosis of previous inadequate treatment and a chronic apical periodontitis secondary to an un-treated canal was made. It was decided to perform a non-surgical endodontic retreatment. An informed consent was taken from the patient.

**Table 1:** Summary of case reports on maxillary lateral incisor with two roots

	**Author**	**Journal**	**Year**	**Associated Developmental Defect**	**Treatment**	**Accessory Root**	**Follow-up**	**Full text**
**1**	**Sykaras [** 5 **]**	OOO	1972	N/A	N/A	Retained	N/A	no
**2**	**Peikoff and Trott [** 6 **]**	JOE	1977	PGG	Extraction	Removed	N/A	yes
**3**	**Christie ** ***et. al.*** ** [** 7 **]**	JOE	1981	PGG	Orthograde RCT of both roots	Retained	48	yes
**4**	**Christie ** ***et. al.*** ** [** 7 **]**	JOE	1981	Dens	Orthograde and Retrograde RCT of Accessory root only	Retained	12	yes
**5**	**Fried and Winter [** 8 **]**	Periodontal Case Rep	1984	N/A	N/A	N/A	N/A	no
**6**	**Peikoff ** ***et. al.*** ** [** 3 **]**	JOE	1985	PGG	Extraction	Removed		yes
**7**	**Vire [** 9 **]**	ooo	1985	N/A	Extraction	Removed	N/A	yes
**8**	**Sabala [** 10 **]**	J Okla Dent Assoc	1986	N/A	N/A	N/A	N/A	no
**9**	**Greenfeld and Cambruzzi [** 11 **]**	OOO	1986	Dens	Orthograde RCT of accessory canal. Main canal not treated	Retained	24	yes
**10**	**Greenfeld & Cambruzzi [** 11 **]**	OOO	1986	Dens	Orthograde RCT and accessory root removed	Removed	12	yes
**11**	**Yoshikawa ** ***et al.*** ** [** 12 **]**	J Osaka Dent Uni	1987	N/A	N/A	N/A	N/A	no
**12**	**Hatton and Ferrillo [** 13 **]**	JOE	1989	N/A	Orthograde RCT of main root and Retrograde RCT of accessory root	Retained	12	yes
**13**	**Fabra-Campos [** 14 **]**	JOE	1990	PGG	Orthograde RCT of both roots	Retained	36	yes
**14**	**Pecora and Santana [** 15 **]**	Braz Dent J	1991	N/A	Orthograde RCT of both roots	Retained	N/A	yes
**15**	**Platt [** 16 **]**	Gen Dent	1995	N/A	N/A	N/A	N/A	no
**16**	**Peix-Sanchez and Minana-Laliga [** 17 **]**	IEJ	1999	2 canals in accessory root	Orthograde RCT of both roots	Retained	11	yes
**17**	**Wei ** ***et al.*** ** [** 18 **]**	J Periodontol	1999	PGG	Orthograde RCT of main canal, accessory root removed, PGG removed by radiculoplasty	Removed	12x7	yes
**18**	**Collins [** 19 **]**	Aus Endod J	2001	No	Incomplete Orthograde RCT	Retained	0	yes
**19**	**Low and Chan [** 20 **]**	Aus Endod J	2004	No	Orthograde RCT for both roots	Retained	6	yes
**20**	**Low and Chan [** 20 **]**	Aus Endod J	2004	Possible PGG	Orthograde RCT for both roots	Retained	6	yes
**21**	**Yavuz ** ***et al.*** ** [** 21 **]**	JOE	2008	No	Orthograde RCT of main root, accessory root removed	Removed	12	yes
**22**	**Venugopal and Srirekha [** 22 **]**	Annals Ess Dent	2010	N/A	Orthograde RCT of main root and accessory root removed	Removed	6	yes
**23**	**Ravindranath ** ***et al. *** **[** 23 **]**	Gen Dent	2011	N/A	N/A	N/A	N/A	no
**24**	**Dexton ** ***et al.*** ** [** 24 **]**	J Conserv Dent	2011	N/A	Orthograde Re Treatment	Retained	24	yes
**25**	**Gandhi ** ***et al.*** ** [** 25 **]**	IEJ	2011	PGG	Orthograde RCT of main canal, accessory root removed, Radiculoplasty and GIC restoration of PGG	Removed	12	yes
								
**26**	**Singh Matta [** 26 **]**	Iran Endod J	2012	Pit over cingulum	Orthograde RCT of both roots	Retained	12	yes
**27**	**Mohan ** ***et al. *** **[** 27 **]**	Contemp clin dent	2012	No	Orthograde RCT of both roots	Retained	N/A	yes
**28**	**Rajput ** ***et al.*** ** [** 28 **]**	Ind J Dent Res	2012	PGG	Orthograde RCT of both canals, restoration of PGG with GIC	Retained	18	yes
**29**	**Lee ** ***et al. *** **[** 32 **]**	Rest Dent Endo	2013	PGG	Orthograde retreatment of main root and Orthograde RCT of accessory root	Retained	6	yes
**30**	**Lee ** ***et al.*** ** [** 32 **]**	Rest Dent Endo	2013	Main canal c shaped	Orthograde RCT of both roots	Retained	6	yes
**31**	**Hoseini and Abbaszadegan [** 29 **]**	J Dent Shiraz Univ Med Sci	2014	Pit over cingulum	Orthograde RCT of both roots	Retained	6	yes
**32**	**Aminsobhani and Meraji [** 30 **]**	J Dent Tehran Uni	2015	N/A	Orthograde Retreatment of main root and orthograde RCT of accessory root	Retained	16	yes
**33**	**Çalışkan ** ***et al.*** ** [** 31 **]**	Iran Endod J	2016	Dens	Accessory root removed only	no	84	yes

*N/A: Not Available, PGG: Palato Gingival Groove, Dens: Dens Invaginatus, GIC: Glass Ionomer Cement*

**Figure 2:**
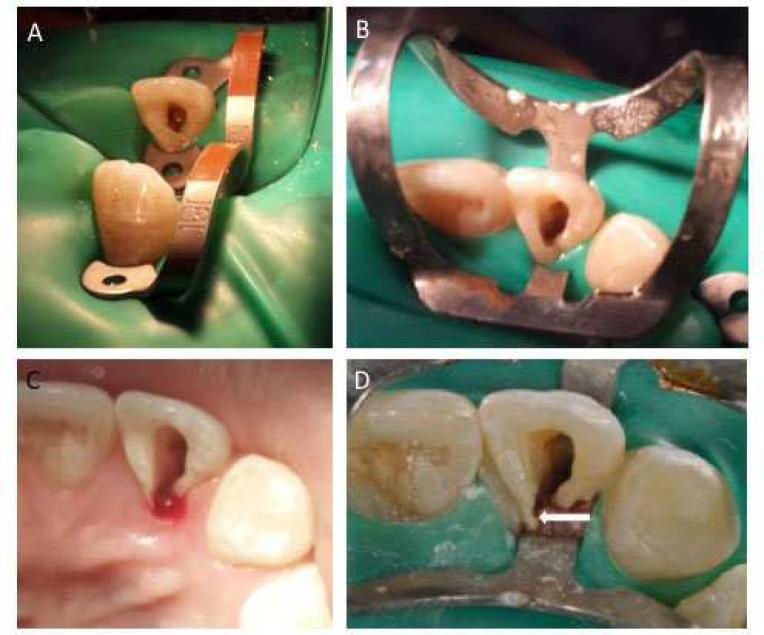
*A)* Initial access after removing restorative material; *B)* Access modified lingually; orifice of accessory root was still not located; *C)* Removal of gingival tissue with a round bur; *D)* Arrow shows the location of accessory orifice on the mesiopalatal aspect

All treatments were performed under a magnification 2.5× loupes and an overhead light source (Tao’s Optics, Nanjing, China). After administering local anesthesia of Lidocaine 2% with 1:100000 epinephrine (Medicaine Houns Co. Ltd, Korea), a rubber dam was placed. Restorative material from access cavity was removed with a round diamond bur (size ISO 001/016 Mani, Japan) and previous root filling was removed with ProTaper retreatment files (Dentsply Maillefer, Ballaigues, Switzerland). However, the orifice of accessory root could not be found with this conventional access. Main canal was filled with calcium hydroxide (Calcipast, Cerkamed, Stalowa Wola, Poland) and access cavity was restored with Cavit (ESPE-Premier, Norristown, PA, USA) and patient was recalled after a week. On subsequent appointments, the access was modified by extending more gingivally to involve the cingulum while some gingival tissue was also removed to expose the cingulum completely (Figure 2C). Attempts to locate the accessory canal often resulted in placing the files into the periodontal ligaments of furcation between the two roots. 

**Figure 3:**
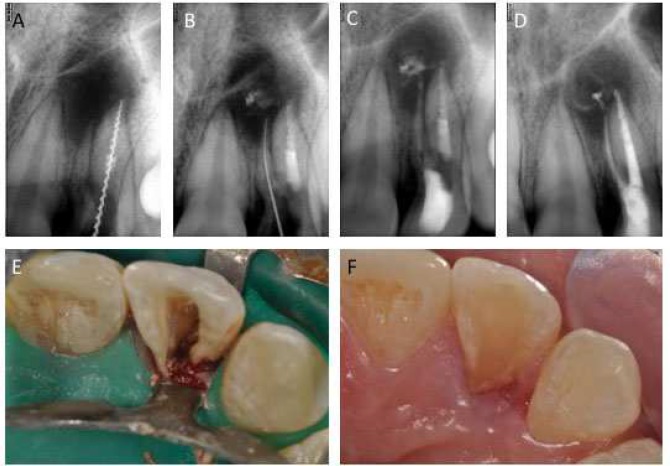
*A)* Working length radiograph for the main canal; *B)* Working length radiograph for the accessory root; *C)* Intra-canal medicament; *D)* Post-obturation radiograph; *E)* Access cavity after obturation; *F)* Restored access cavity

After much efforts, the canal orifice was eventually located on mesio palatal aspect of modified access cavity with a mesially angulated #10 K-file (SybronEndo Corporation, Orange, CA, USA) (Figure 2D). Working length was established with electronic apex locator (Locapex Five, Ionyx, Blanquefort, France) and verified radiographically. Canal preparation was performed with ProTaper rotary NiTi instruments according to manufacturer instructions (Dentsply Maillefer, Ballaigues, Switzerland). Canal was copiously irrigated between the use of each file with 5.25% sodium hypochlorite (CHLORAXID 5.25%, Cerkamed, Stalowa Wola, Poland). Subsequently, both canals were dried and calcium hydroxide was placed and access cavity closed with a temporary restorative material (Cavit™, 3M, Maplewood, Minnesota, USA) (Figure 3C). 

Patient reported after three weeks. By this time all the symptoms had subsided. It was decided to obturate both the canals. After local anesthesia and rubber dam isolation, canals were irrigated with sodium hypochlorite to remove the dressing and dried with paper points. Canals were obturated with warm vertical technique using an obturation device (Elements Obturation Unit, Kerr Corporation, Orange, CA, USA) and a resin based sealer (AH-Plus, Dentsply Maillefer, Ballaigues, Switzerland) (Figure 3D). Access cavity was restored with a flowable composite resin (Filtek flow, 3M, Maplewood, Minnesota, USA). At this point, the surgical correction of palatogingival groove was deferred since some tooth structure was already sacrificed in attempts to locate the accessory orifice. 

**Figure 4:**
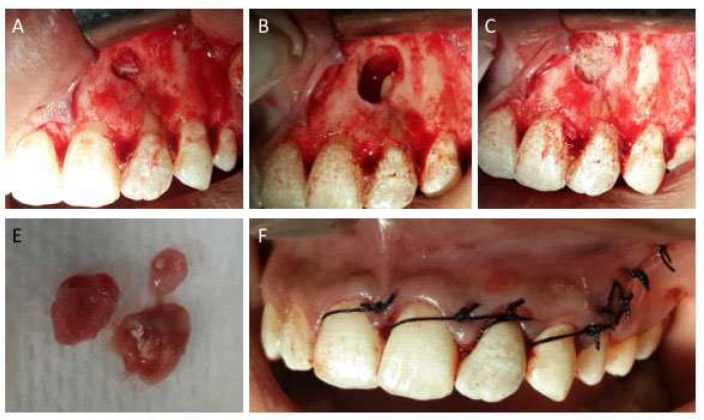
*A)* View of the surgical field reveals absent cortical plate at apical area, a bony dehiscence is also present on the lateral incisor; *B)* Granulation tissue completely removed; *C)* Bony cavity filled with bone graft; *D)* The removed tissue; *E)* Sling sutures were used

**Figure 5:**
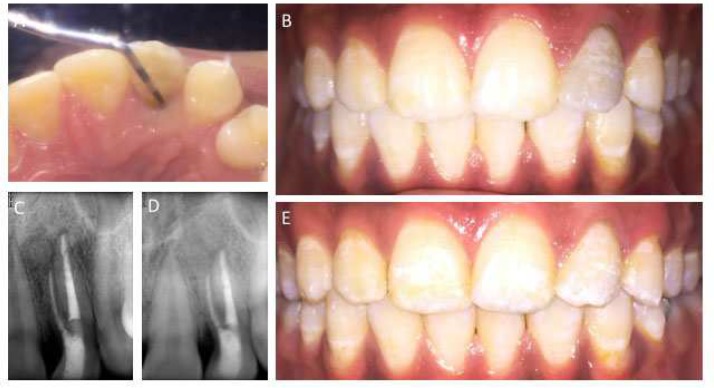
*A)* 2 year follow-up, reduced probing depth; *B)* Discoloration of the crown was present; *C)* 1 year follow-up radiograph; *D)* 2 year follow-up radiograph; *E)* Walking bleach was used to remove discoloration

Patient returned after a month for recall visit and complained of recurring pain and pus discharge. At the time of visit, no sinus tract was detected. Based on symptoms of pain, it was decided to perform surgical retreatment. On next visit a full thickness mucoperiosteal flap was raised with one relieving incision involving the distal papilla of canine (Figure 4A). A defect in buccal plate could be seen located around the apex of accessory root. After further osteotomy of buccal plate, the periapical lesion was enucleated. The lesion was found to have no attachment to the either root and was removed quiet easily (Figure 4B). Due to this fact we decided not to perform root end resection. The bony window was filled with an irradiated allograft (Rocky Mountain Tissue Bank, S. Peoria, Aurora, Colorado, USA) and covered with a resorbable collagen membrane (BioMend, Warsaw, Indiana, USA) (Figure 4C). Sling suturing technique using 3.0 silk sutures (Glysilk, Huailyin Medical Instruments Factory, China) was used for primary closure and simple interrupted sutures were used for relieving incision (Figure 4F). Post-operative instructions were given and patient was recalled. The stitches were removed after a week. On a 2-year follow-up patient reported complete absence of symptoms, reduced periodontal probing depth on palatal aspect and radiographic signs of complete healing but with mild discoloration of clinical crown (Figures 5A and B). The discoloration was treated with a walking bleach technique using sodium perborate powder.

## Discussion

Management of a maxillary lateral incisor with two separate roots has been reported previously and an exhaustive summary is presented (Table 1). We excluded those case reports from this list that presented with two canals in the same root, fusion with supernumerary or standalone dens invaginatus without presence of accessory root [35-37]. It is evident from this review that either the accessory root was root treated and retained or extracted. The decision for extraction may be necessary if the accessory root is not patent. However, every effort must be spent to retain it if a radiographically visible canal is present. Removal of this root might result in an un-esthetic outcome, *i.e.* loss of interdental papilla due to loss of interdental bone height. It has been reported previously that position of interdental papilla is influenced by the height of interdental bone [38]. In our case, the loss of papilla was avoided by retention of accessory root. Yavuz *et al. *[39] reported slight loss of papilla in their case where the accessory root was removed due to lack of patency.

In the present case report, endodontic treatment failure occurred because of lack of knowledge of the two roots as mostly maxillary lateral incisor is associated with a single root [40]. This led to persistent pain and infection in the tooth. When pre-operative radiographs with different angulations were taken, two roots were clearly visible. In such cases cone-beam computed tomography (CBCT) is very useful tool but due to the unavailability in our set up, it was not used.

The presence of palatogingival groove was probably not related to the patient’s symptoms. We found increased probing depth on palatal surface at the area of bifurcation. However, since the probing depth returned to normal after treatment completion and remained stable at 2-year follow-up, it is likely that the groove did not contribute to the periodontal defect. Withers also reported that every groove is not responsible for periodontal destruction [41]. However, Wei *et al.* [18] reported that the presence of furcation in a birooted incisor further complicates the treatment outcome in the presence of a palatogingival groove. Furthermore, it is likely that in our case the groove terminated at cemento-enamel junction similar to the case reported by Wei *et al.* [18]. Since no flap was raised on the palatal aspect, this finding could not be confirmed. 

The orifice of accessory root was found to be entirely dissociated with the pulp chamber of primary canal. It has been suggested that the palato-gingival groove was a failed attempt to form a second root and it may lead to formation of an entirely distinct pulp space [42]. In the absence of a three dimensional imaging, exact location of the orifice may be extremely difficult to find. An operating microscope may aid in location; however, it was not available. Instead, a magnification 2.5× loupes and an overhead light source was used. The access outline of our case seems very similar to that reported by Low *et al*. [20].

The question whether tooth was associated with a true cyst or pocket cyst remains unanswered as the specimen for biopsy was lost.

## Conclusion

The successful negotiation of accessory root of maxillary lateral incisor and its surgical treatment resulted in a favorable outcome for the patient after 2 years of un-eventful healing period. This case points towards the importance of pre-operative assessment of number of roots and canal configuration with different angulated radiographs and need of CBCT in special circumstances which are all prerequisite for successful endodontic treatment. 
